# Autonomic nervous system and hypothalamic–pituitary–adrenal axis response to experimentally induced cold pain in adolescent non-suicidal self-injury – study protocol

**DOI:** 10.1186/s12888-015-0544-4

**Published:** 2015-07-07

**Authors:** Julian Koenig, Lena Rinnewitz, Marco Warth, Michael Kaess

**Affiliations:** 1 Department of Child and Adolescent Psychiatry, Centre for Psychosocial Medicine, University of Heidelberg, Heidelberg, Germany; 2 Department of Psychology, The Ohio State University, Columbus, OH USA; 3 School of Therapeutic Sciences, SRH University Heidelberg, Heidelberg, Germany

**Keywords:** Non-suicidal self-injury, Pain sensitivity, Autonomic nervous system, Hypothalamic–pituitary–adrenal axis

## Abstract

**Background:**

Adolescent non-suicidal self-injury (NSSI) is associated with altered sensitivity to experimentally induced pain. Adolescents engaging in NSSI report greater pain threshold and pain tolerance, as well as lower pain intensity and pain unpleasantness compared to healthy controls. The experience of pain is associated with reactivity of both the autonomic nervous system (ANS) and the hypothalamic–pituitary–adrenal (HPA) axis. However, previous research has not yet systematically addressed differences in the physiological response to experimentally induced pain comparing adolescents with NSSI and age- and sex-matched healthy controls.

**Methods/Design:**

Adolescents with NSSI and healthy controls undergo repeated painful stimulation with the cold pressor task. ANS activity is continuously recorded throughout the procedure to assess changes in heart rate and heart rate variability. Blood pressure is monitored and saliva is collected prior to and after nociceptive stimulation to assess levels of saliva cortisol.

**Discussion:**

The study will provide evidence whether lower pain sensitivity in adolescents with NSSI is associated with blunted physiological and endocrinological responses to experimentally induced pain compared to healthy controls. Extending on the existing evidence on altered pain sensitivity in NSSI, measured by self-reports and behavioural assessments, this is the first study to take a systematic approach in evaluating the physiological response to experimentally induced pain in adolescent NSSI.

**Trial Registration:**

Deutsche Register Klinischer Studien, Study ID: DRKS00007807; Trial Registration Date: 13.02.2015

## Background

Non-suicidal self-injury (NSSI) is the self-directed act of harming one’s own body tissue (i.e., cutting or burning) without suicidal intent [1]. According to a recent meta-analysis, the prevalence for NSSI in non-clinical samples is 17.2 % among adolescents, 13.4 % among young adults, and 5.5 % among adults [2]. A characteristic feature of NSSI is an alteration of sensitivity to experimentally induced pain, that is also found in other psychiatric conditions associated with self-injurious behavior (SIB, i.e., the intentional, self-directed act of injuring one’s own body tissue by cutting, burning etc. regardless of the suicidal intent) such as borderline personality disorder (BPD) or suicidal ideation [3]. Individuals engaging in SIB report greater pain threshold and pain tolerance in addition to lower pain intensity and pain unpleasantness in comparison to healthy controls (for a complete review on the literature on pain sensitivity and SIB see Koenig J, Kaess M, Thayer JF. A Meta-Analysis of Self-Injurious Behavior and Sensitivity to Experimentally Induced Pain. Submitted).

Recurrent NSSI is a core feature of BPD [4]. BPD is a severe personality disorder that is often characterized by a pervasive pattern of impulsivity, emotional instability, interpersonal dysfunction and a disturbed self-image [5], and has an approximate suicide rate of 8–10 % [6]. The disorder usually emerges during adolescence and continues into adulthood – it is regarded as both a dimensional construct and a disorder, and has recently been confirmed as a diagnosis for adolescents in the new Diagnostic and Statistical Manual of Mental Disorders (DSM-V, [4]). Borderline personality pathology underlies repetitive self-harm in a substantial proportion of young individuals. More than 30 % of retrospective reports from adults with BPD indicate childhood-onset of self-injury, with another 30 % who indicate adolescent-onset [7]. BPD can be diagnosed in the majority of female adolescent inpatients with repetitive NSSI [8], and the number of BPD criteria met is predictive of whether or not an adolescent has engaged in SIB [9].

### Autonomic nervous system and hypothalamic–pituitary–adrenal axis response to acute pain

The experience of acute pain leads to a variety of physiological responses. The reactivity of the autonomic nervous system (ANS) to experimentally induced pain is best described by an increase of sympathetic activity and a decrease of parasympathetic activity. Reactivity of the ANS has been found to be associated with pain sensitivity [10] and to be a significant predictor of pain tolerance [11]. Sympathetic nervous system activity increases with more intense and painful stimulation [12].

On the other hand, nociception is associated with response of the hypothalamic–pituitary–adrenal (HPA) axis. Painful stimulation typically leads to an increase in cortisol release [13]. However, some studies do not report an increase in cortisol due to painful stimulation [14]. A greater cortisol awakening response is associated with greater sensitivity (i.e., ratings of pain intensity and unpleasantness) to experimentally induced pain [15, 16]. Sex differences have been reported in children, such that cortisol levels are positively associated with increased pain tolerance in boys and increased pain sensitivity in girls [17]. Greater pain reports following nociceptive stimulation are associated with greater increases in cortisol response [18, 19]. To conclude briefly, both – the ANS and the HPA axis – are distinct physiological systems that are involved in the human response to experimentally induced pain and are related to pain sensitivity.

### Autonomic nervous system and hypothalamic–pituitary–adrenal axis functioning in NSSI

Compared to healthy controls, both ANS and HPA axis activity and reactivity to stress seem to be altered in individuals with NSSI and/or BPD [20, 21] but the data are inconsistent. BPD patients show a significantly higher startle response compared to healthy controls [22]. Other findings suggest lower ANS reactivity to negative pictures in BPD patients [23]. It has been found that imagery of disorder-specific scripts can lead to a potentiated startle responses and increased autonomic arousal in BPD patients compared to healthy controls [24]. However, other studies found that BPD patients do not differ from healthy controls in their ANS responses to standardized photographic slides compared to healthy controls [25–27]. No previous study has systematically investigated ANS response to experimentally induced pain in SIB.

Patients with BPD have shown a higher salivary cortisol awakening response and higher total daily cortisol levels compared to healthy controls [28]. Other studies suggest that basal cortisol levels are only enhanced in BPD patients with comorbid post-traumatic stress disorder [29], in line with previous research, suggesting that HPA dysfunctions in BPD appears to be related to childhood trauma rather than psychopathology in adulthood [30]. Experimental studies found that individuals with BPD showed a hyperresponsiveness of the HPA axis using a combined dexamethasone/corticotropin-releasing hormone test, as indicated by enhanced corticotropin and cortisol response [31]. Such hypersensitivity is further supported by another study using dexamethasone as an endogenous agent [32]. However, these findings were not replicated by a later study [33] and the usefulness of such testing in BPD has been questioned [34]. More recently, experimental studies using a laboratory stress paradigm (Trier Social Stress Test) demonstrated an attenuated cortisol response in adults with BPD [35], and similarly, in adolescent patients with repetitive NSSI [36]. Since the experiences of early chronic stress has previously been linked to a pattern of HPA axis hyperresponsiveness in early life, a switch to hyporesponsiveness of the central and/or peripheral cortisol release may occur later in life [37]. However, previously no study investigated HPA axis response to experimentally induced pain in NSSI.

### Objective

NSSI is associated with altered sensitivity to experimentally induced pain. There is evidence that ANS and HPA reactivity is different in NSSI compared to healthy controls – in particular in patients with BPD – although existing studies are not conclusive. ANS and HPA axis activity is associated with pain sensitivity to experimentally induced pain, and ANS and HPA axis are reactive to nociceptive stimulation. However, no study has linked such findings by exploring ANS and HPA axis reactivity to experimentally induced pain in NSSI. Thus, the present study aims to investigate ANS and HPA axis response to experimentally induced pain in NSSI patients compared to age- and sex-matched healthy controls. We hypothesize, that adolescents with NSSI exhibit decreased ANS and HPA reactivity to experimentally induced pain compared to healthy controls. Specifically, we hypothesize that the ANS response in adolescents with NSSI will show blunted sympathetic but greater parasympathetic activity in response to painful stimulation.

Addressing potential differences in the physiological and endocrinological response to pain in individuals engaging in SIB might help to enlighten mechanisms underlying the well know differences on self-reports and behavioral measures of pain sensitivity. Previous research on pain response in SIB did not systematically address this autonomic component and predominantly focused on the motivational and emotional domains. However, ANS and HPA responses to pain are well known to shape the individual pain experience and thus might further contribute to altered pain sensitivity in individuals engaging in SIB. Secondly, each participant will undergo a repeated stimulation after a 25-min washout period to address potential differences in the habituation to painful experiences.

## Methods/Design

The study is an experimental laboratory trial utilizing a case control-group design, comparing adolescents with NSSI to age- and sex-matched healthy controls. A priori power analysis based on previous studies and meta-analysis of reported group effects (﻿Koenig J, Kaess M, Thayer JF. A Meta-Analysis of Self-Injurious Behavior and Sensitivity to Experimentally Induced Pain. Submitted) helped to determine the aimed sample size of 30 participants per group.

### General procedures

Adolescents with NSSI and age- and sex-matched healthy controls are enrolling in the study. After recruitment, participants undergo an initial baseline assessment and diagnostic screening. The experimental procedure comprises the repeated stimulation (twice) with a cold pain stimulus to assess pain sensitivity. Physiological recordings of ANS activity are taken throughout painful stimulation. Endronicological assessments of salivary cortisol, indexing HPA axis reactivity, occurs before and after each trial of painful stimulation. Additionally, we ask participants to complete several self-report questionnaires to control for potential covariates of pain sensitivity and physiological reactivity. All clinical and baseline assessments are carried out at the *Ambulanz für Risikoverhalten & Selbstschädigung* (AtRiSk) at the Clinic of Child and Adolescent Psychiatry, Centre of Psychosocial Medicine, University of Heidelberg. The experimental procedures are carried out at the Lab for Pain Research, at the School of Therapeutic Sciences, at the SRH University, Heidelberg, Germany.

### Ethical approval and recruitment of participants

We received ethic approval to conduct the study outlined in this research protocol by the Ethical Committee of the Medical Faculty, Heidelberg University, Germany (study ID S-471/2013). Participants (aged 12 to 17 years) in the NSSI group (n = 30) are recruited consecutively from the specialized outpatient clinic for risk-taking and self-harm behaviour (AtRiSk; *Ambulanz für Risikoverhalten & Selbstschädigung*) at the Clinic of Child and Adolescent Psychiatry, Centre of Psychosocial Medicine, University of Heidelberg. For the healthy control group (n = 30), age- and sex-matched participants are recruited via public advertisements according to a standardized procedure within the clinic. Recruitment is restricted to females given the known sex differences in prevalence of NSSI and in HPA axis response to stress [38]. Information material is prepared to inform adolescents and their relatives about the aim of the study. Information material is available for potential participants of the healthy control group and the NSSI group. We obtain written informed consent from adolescents and their first-degree relatives prior to inclusion in the study. Participants receive 30€ for participating in the study.

### Clinical diagnostics and baseline assessment

We recruit adolescents with NSSI, reporting NSSI behavior on at least five days during the past 12 months, at the AtRiSk clinic who undergo a standardized psychological assessment. Aside from the assessment of basic socio-demographic variables, assessments include: *the German version of the Self-Injurious Thoughts and Behavior Interview* (SITBI-G) for the detailed assessment of NSSI, the German version of the *Mini-International Neuropsychiatric Interview for Children and Adolescents* (M.I.N.I- KID 6.0); the respective part of the German version of the *Structured Clinical Interview for DSM-IV-Axis II* (SKID-II) to assess borderline personality disorder; the *Children’s Global Assessment Scale* (C-GAS), a German depression inventory for children and adolescents (*Depressions-Inventar für Kinder und Jugendliche*, [Depression Inventory for Children and Adolescents] DIKJ); and the *Childhood Experience of Care and Abuse Questionnaire* (CECA.Q).

The SITBI-G [39] is a semi-structured interview for the assessment of the presence, frequency, and characteristics of a wide range of self-injurious thoughts and behaviors, including suicidal ideation, suicide plans, suicide gestures, suicide attempts, and NSSI, and shows excellent psychometric properties. Clinical diagnoses are assessed using the M.I.N.I- KID [40] and one part of the SKID-II [41]. The M.I.N.I.-KID is a short structured diagnostic interview for DSM-IV and ICD-10 psychiatric disorders for children and adolescents aged 6 to 19 years. The borderline- personality disorder portion of the SKID-II interview is used.

The C-GAS [42] is a widely used numeric rating scale (1 through 100) to rate the general functioning of children under the age of 18. The DIKJ [43] is a German screening inventory for depressive symptoms in children and adolescents, based on the Children’s Depression Inventory [44]. It comprises 26 items that are rated on three-point scales and aggregated into a total score. It has shown to have excellent psychometric properties [43]. The CECA.Q [45] obtains information on current relationships and retrospective information on family arrangements and parental loss during childhood focusing on the life period prior to the age of 17. Screening questions assess physical and sexual abuse and two scales measure antipathy and neglect by the mother and father figure. The German version of the CECA.Q [46] is used.

Participants recruited for the control group, also complete screening questions of the SITBI, are rated on the C-GAS, complete the DIKJ and the CECA.Q. In addition, they complete the structured clinical interview (SCID-N/P [47]), for DSM-IV-TR non-patient edition, to control for the presence of any psychiatric disorder.

### Cold pain stimulation

Cold pain sensitivity is assessed twice via a previously evaluated [48] cold pressor task (CPT) procedure. Participants are asked to immerse their hand up to the wrist in an acrylic glass tank with circulating water (three floor pumps Conrad Electronic GmbH AP-333, water flow: each 200 l/h) to prevent local warming. The immersion of the dominant or non-dominant hand is cross-randomized across trials. There is a 25-min wash-out period between trials. Water temperature of the CPT is kept at 4 °C and controlled constantly with a chilling device (Resun CL 450) and water pump (Conrad Electronic GmbH Item no. 55 16 73, 1400 l/h) and measured with two digital underwater thermometers (range of temperature: −40 to 150 °C) with a built-in 100mAh lithium battery (Thermograph TEMPr B, B080169) at different spots (chiller inflow, chiller outflow). Water temperature is recoded continuously (every second) by each thermometer and transferred to a PC-based software via USB hub. Participants are instructed to keep their hand open (rather than closed, fist position) while it is in the water. Before the immersion, the participants are told to keep the hand in the water until cold pressor pain turns intolerable, with a cutoff time of 4 min. The latencies to the first pain sensation (*pain threshold*) and to the intolerable pain (*pain tolerance*) are measured with a stopwatch in seconds. Ambient temperature was recorded before the start of the procedure to control for potential covariates. Prior to nociceptive stimulation a check-list asking for potential covariates of physiological response due to cold stimulation and reasons for exclusion from the study is completed. Participants are asked for the presence of any cardiovascular disease, hypertension, past cramp attacks or blackouts, chilblains, known Reynaud syndrome, open wounds, or fracture of the hands. Their weight and height is recorded to calculate their body mass index (BMI) that is an important covariate of ANS activity [49]. We ask them to indicate their dominant hand and if they previously took part in a study comprising experimental painful stimulation, specifically using the CPT.

### Autonomic nervous system and endocrinological assays

Hear rate (HR) is continuously recorded throughout the procedures with a Polar RS800CX portable device using a transmitter consisting of a stable polyamide case with electrodes attached to an elastic belt fixated to the chest of participants. Chronotropic control of the heart is achieved via the complex interplay of the sympathetic (SNS) and the parasympathetic (PNS) branches of the ANS. HR is under tonic inhibitory control (PNS dominance over SNS influences) [50], and the PNS modulation of the HR is fast (timescale of milliseconds) and short-lived, while SNS effects are slow on the timescale of seconds [51]. The recording and analysis of the sequence of time intervals between adjacent heartbeats – the inter-beat-interval (IBI in milliseconds) - is therefore the basis for the calculation of all the measures of heart rate variability (HRV). HRV is a reliable and readily available measure of parasympathetic nervous system activity that typically decreases after painful stimulation [52]. The Polar RS800CX is capable to produce IBIs at sampling frequency of 1000 Hz, providing a temporal resolution of 1 ms for each R–R interval. Device-specific software (Polar ProTrainer 5) is used to transfer recordings to a PC. IBI data (.txt files) is exported and HRV is analyzed using Kubios HRV (Biosignal Analysis and Medical Imaging Group, University Kuopio, Finland, Version 2.0) [53]. Different indices of HRV are subsequently derived for further analysis. Given our particular interest in vagal activity, the square root of the mean squared difference of successive NN intervals (RMSSD, *ms*), the proportion of pairs of successive NNs that differ by more than 50 ms (pNN50, %), and the spectral power expressed as normalized units of the high-frequency (HFn.u.; 0.15–0.4 Hz) band derived using a autoregressive algorithm are obtained. In case of screwed distributions, HRV data will be log-transformed for further statistical analysis [54]. We derive HR and HRV for different time segments in the course of the procedure. There is a 5 min baseline recording before hand-immersion in the CPT and a 10 min post-line recording after hand-removal from the CPT. In addition to the continuous assessment of HR and HRV, blood pressure (BP) is measured for a total of four times, initiated at least 5 min before each CPT and immediately after hand removal using an automated digital device (Braun ExactFit™ 3 BP6000). We use an estimation of systolic and diastolic BP values, in addition to mean arterial pressure (MAP), for further analysis.

We assess HPA axis response using salivary samples. Saliva cortisol is a reliable and valid proxy of free plasma cortisol levels [55, 56], sensitive to changes in HPA axis activation due to stressors [55] such as experimentally induced pain [57], and has previously been utilised in numerous studies with children and adolescents [58–60]. We collect four salivary samples; one prior to each CPT and one following 15 min after hand removal. Collection of saliva samples is carried out  by having participants chew on a cotton role (Salivettes; Sarstedt, Numbrecht, Germany) for 2 min. After the collection of salvia, samples (at least 1000 μl salvia) are labelled with the participant ID and time-point of assessment, and then are directly deep-freezed at −20 ° C and stored until assay. Cortisol will be analysed at the Institute of Pharmacology of the Medical Faculty Heidelberg (Steroid Lab). For preparation, samples will be centrifugated for 7 min at 3000 rpm and 100 μl salvia per participant will be used for assay. The assay for the determination of salvia cortisol and serum cortisol is a special in-house assay with extraction and subsequent radioimmunoassay (RIA) developed by the Steroid Lab at the Institute of Pharmacology of the Medical Faculty Heidelberg. The range of the standard curve is from 2,5 to 5000 pg including a limit of detection of 5 pg/probe. The intra-assay coefficient of variation is below 10 % and inter-assay coefficient of variation is below 15 %.

### Self-report questionnaires and additional scales

The German version [61] of the short form McGill Pain Questionnaire (SF-MPQ [62]), is administered after the each CPT. The SF-MPQ was derived from the McGill Pain Questionnaire (MPQ [63]), and allows quantitative, multidimensional pain ratings to be obtained in a brief period of time. The SF-MPQ consists of 15 descriptors (11 *sensory*, 4 *affective*) that are rated on a 4-point intensity scale from zero (“*none*”) to three (“*severe*”). Three pain scores (sensory, affective, and total descriptors) are derived from the sum of the intensity rank values of the words chosen for descriptors. Pain intensity on hand removal is assessed on a 10 cm visual analogue scale (VAS) from zero to ten, and the overall pain experience is assessed by one descriptor (“*no pain*”, “*mild*”, “*discomforting*”, “*distressing*”, “*horrible*”, or “*excruciating*”). In addition, participants complete the Quick Inventory of Pain Symptoms (QIPS) on arrival at the pain laboratory that assesses pain symptoms in 5 major locations during the past week [64]. Finally, participants rate three self-developed scales on arrival at the pain laboratory and after each CPT. Participants rate their momentary mood *(“Wie geht es dir gerade?”*), their felt tension *(“Wie hoch ist deine Anspannung?”*), and their body-awareness *(“Wie sehr spürst du dich gerade?”*), each on a 10 cm VAS ranging from 0 to 100.

### Data analysis

We will assess group differences in cardiovascular and endocrinological responses to experimentally induced pain, by comparing measures taken before and after pain induction as primary study outcome. Analysis will be conducted using a repeated measures approach exploring the main and interaction effects of the time of measurement (i.e., pre, post painful stimulation) and group (i.e., NSSI, healthy controls) on all dependent variables (i.e., cortisol, RMSSD, MAP) controlling for potential covariates (i.e., BMI). Further analysis will address group differences on reports of pain threshold and pain tolerance, as well as self-reports of pain intensity. Exploratory analysis will address changes in self-rated mood, tension, and body-awareness comparing assessments taken before and after pain induction and their association to physiological reactivity as well as self-reports of pain experience.

## Discussion

The present study aims to investigate differences in ANS- and HPA-axis response to experimentally induced cold pain in adolescents with NSSI compared to a sample of age- and sex-matched healthy controls. Differences on pain sensitivity in induced thermal cold pain in SIB have previously been studied [65–74]. However, none of the previous studies aimed to investigate differences in the physiological response to nociception using measures of ANS or HPA axis activity, that are known to differ in individuals fulfilling clinical criteria for NSSI and/or BPD.

We decided to use cold pain stimulation via the CPT as nociceptive stimuli, as it is a well-established experimental procedure [75] to induce cold pain and is ethically acceptable also in the use with younger participants [76–78], developed originally as a clinically indicative cardiovascular test, and thus, produces a reliable ANS response [79–82]. Different temperatures are proposed for the use of the CPT [83]. Studies on NSSI used temperatures as high as 10 °C [65] to as low as 0 °C [71] – all producing significant differences between individuals engaging in SIB and healthy controls. We decided to use a temperature of 4 °C, as it previously produced the most reliable results using this apparatus [48]. Participants of the present study receive repeated stimulation with the CPT, to control for lateral dominance and differences between both hands (dominant vs. non-dominant) that have previously been supported by some studies [84–87]. Furthermore, this will allow for investigating potential differences in the short-term habituation to pain in NSSI compared to healthy controls.

We assess ANS response to painful stimulation using measures of HR, HRV, and BP. One might critically note that while we use a portable device to record HR and HRV, a stationary assessment of ANS activity (e.g. using a 3-lead electrocardiogram), is to be preferred. However, as we were forced to compromise, we used a device comparable to others, showing good validity and appearing to be reliable [88–90]. Furthermore, the device has been frequently used within other studies (e.g. [91, 92]).

The current and ongoing study will provide evidence whether less pain sensitivity in adolescents with NSSI is also associated with blunted physiological and endocrinological responses to experimentally induced pain compared to healthy controls. Existing evidence on altered pain sensitivity in NSSI is largely based on self-reports of pain intensity and pain unpleasantness as well as behavioural measures of pain threshold and tolerance. This is the first study to take a systematic approach, and evaluate ANS- and HPA-axis response to experimentally induced pain in adolescent NSSI, extending the current perspective on altered pain sensitivity in NSSI. Data collection has an expected completion period of late summer, 2015. A 1-year follow-up on the participants is planned to further explore the temporal stability of alterations in pain sensitivity in those engaging in NSSI, reflected by psychophysiological and endocrinological responses to experimentally induced pain.

### Trial status

The study is ongoing and the first 20 participants have been recruited.

## Abbreviations

ANS: Autonomic nervous system
BMI: Body mass index
BP: Blood pressure
BPD: Borderline personality disorder
C-GAS: Children’s Global Assessment Scale
CECA.Q: Childhood Experience of Care and Abuse Questionnaire
CPT: Cold pressure task
DIKJ: Depressions-Inventar für Kinder und Jugendliche [Depression Inventory for Children and Adolescents]
DSM: Diagnostic and Statistical Manual of Mental Disorders
HF: High-frequency
HPA: Hypothalamic–pituitary–adrenal
HR: Heart rate
HRV: Heart rate variability
IBI: Inter-beat-interval
M.I.N.I- KID: Mini-International Neuropsychiatric Interview for Children and Adolescents
MAP: Mean arterial pressure
MPQ: McGill Pain Questionnaire
NSSI: Non-suicidal self-injury
PNS: Parasympathetic nervous system
QIPS: Quick Inventory of Pain Symptoms
SF-MPQ: Short form of the McGill Pain Questionnaire
SIB: Self-injurious behavior
SITBI-G: German version of the Self-Injurious Thoughts and Behavior Interview
SKID-II: Structured Clinical Interview for DSM-IV-Axis II
SNS: Sympathetic nervous system
VAS: Visual analogue scale
